# Spirituality and Psychiatry

**Published:** 2004

**Authors:** Dinesh Bhugra, Thomas R Osbourne

**Affiliations:** 1 * PO Box 25, HSRD, Institute of Psychiatry, De Crespigny Park, London SE5 8AF. d.bhugra@iop.kcl.ac.uk; 2 PO Box HSRD, Institute of Psychiatry De Crespigny Park, London SE5 8AF


*Indian Journal of Psychiatry, 2004, 46(I)5-6*

### Spirituality and Psychiatry

**Dinesh Bhugra*[1] Thomas R Osbourne[2]**

### Introduction

Often religion and spirituality are used interchangeably. Spirituality may or may not include religion, which is related to an organized institution. Spirituality on the other hand is the transcendent relationship between the person and a higher being, a quality that goes beyond a specific religious application (Peterson & Nelson 1989). Both religion and spirituality offer a sense of meaning and purpose in life and help keep believers in relationship to the unknown and unknowable (Lukoff et al 1995).

Why should psychiatrists be interested in spiritual values and religious lives of their patients? First and foremost, it is essential to know what values are forming the individual's being and this moves the biopsychosocial model of aetiology and further management. The discrepancy between the Western Cartesian models of mind body dichotomy and the Ayurvedic models where mind body environment and even seasons are interlinked together needs to be understood if the clinicians wish their patients to work with them. Such a mind- body dualism has led to various academics to allege that somatization is somehow an inferior way of expressing distress. Secondly the role of religion and spirituality in developing models of locus of control has to be understood. If the locus of control for a psychiatric condition is seen as external, the individual (patient) is not blamed and the acceptance of distress may well be easier. Thirdly, cultural psychiatry embraces aspects of social and cultural identity of individuals that are then related to the development and management of distress. Although religion has been seen as opium for the masses by Karl Marx and as universal obsessional neurosis by Freud behaviourists, cognitive behaviour therapists and others have often ignored this impatient aspect in the lives of many of our patients.

### Relationship between Psychiatry and Spirituality

The negative view of religion and spirituality held by some psychiatrists is based on a number of factors. A key factor is seeing religion as primitive, untestable, unverifiable and unscientific especially when psychiatry has been struggling to establish itself as a scientific discipline, which can prove or disprove hypotheses. Psychiatry has chosen to ignore religion as a possible source of strength and well-being and instead focus is on its obsessionality. Religion and spirituality have been seen as pathological especially when the presence of religious and spritual feelings has been construed as abnormal. Patients in view of their religious and spiritual feelings may try and hide these because they feel a lack of sympathy and opprobrium from the clinicians. Furthermore, psychiatrists and other mental health professionals are not trained to deal with the religious and spiritual issues because they feel that they may be working outside the limits of their capability. Lukoff et al (1995) proposed a new diagnostic category – psycho-religious or psychospiritual problems which was accepted by the task force on DSM – IV as Religious or Spiritual Problems which defines these as …" The category can be used when the focus of clinical attention is a religious or spiritual problem. Examples include distressing experiences that involve loss or questioning of faith, problems associated with conversion to new faith…." One of the key problems with such an approach is medicalization of what could be construed as "normal" loss of faith or questioning. There is evidence in the literature to indicate that some patients with psychosis especially in the prodromal period may seek to change their faith either to find stability or start afresh with a new group of acquaintance (Bhugra, 2002).

### Role of Culture and Spirituality

Culture provides a set of meanings and beliefs which give meaning to the way an individual functions and also incorporates religious beliefs and religious rituals. The cultural identity of the individual incorporates their religious and spiritual beliefs. The worldview of individuals will include religious and spiritual beliefs and it has been noted that if the therapist and the patients' worldview diverge too significantly then the engagement for therapy and therapeutic adherence are likely to be affected. The relationship between the individual's religious beliefs may influence the way the patients and their carers approach treatment. The interaction between culture and spirituality is also at multiple levels and patients may have cultural values without religious or spiritual values. Culture determines psychopathology and patients therefore may

*[1]PO Box 25, HSRD, Institute of Psychiatry, De Crespigny Park, London SE5 8AF. Email : d.bhugra@iop.kcl.ac.uk

[2]PO Box HSRD, Institute of Psychiatry De Crespigny Park, London SE5 8AF

*Corresponding Author present with symptoms, which are understandable only in the context of the individual's culture and religion. Religion and spiritual values influence mental health and beliefs about mental illness and its treatment will be affected by religious and spiritual values e.g. some religious groups will refuse to take capsules made from gelatine. The types of religious problems, the clinicians may come across are loss or questioning of faith, conversion to a new religion, intensification of adherence to beliefs and practices and joining new religious movements and cults. The spiritual problems may include possession or trance states, mystical experiences, near death experiences and spiritual emergencies. Using meditation and yoga, as reflections of spiritual goal attainment have become commonplace and again the clinician must take the intensity of these procedures into account. Obsessive-compulsive rituals may from part of spiritual reawakening.

### How to assess spirituality

Religious history which includes the faith patient follows, rites and rituals they perform, religious taboos either in diet or dress they follow will give a good start for the clinician to explore. Good mental health may well be associated with social support, religious ideas, feelings, experiences, orientation and worldview and any assessment must include some of these. Both medicine (and its practice) and religion have common values of humanitarian and moral purpose. Science without religion can be destructive and religion without science can become superstition (Feibelman 1096). A clear cooperation and assessment of individual's needs is essential in ministering to the total need of the person and any assessment therefore must focus on the holistic approach.

In differentiating psychopathology from normal religious and spiritual beliefs Greenberg and Witzum (1991) have proposed that psychotic episodes are more intense than normative religious experiences, are often terrifying and preoccupying for the individual, contain special messages from significant religious figures and are accompanied by deterioration in social functioning.

The clinician's response to these experiences can determine whether the experience is integrated and used as a stimulus for personal growth or whether it is used as a repression phenomenon indicating mental instability. The clinicians can use these phenomena in positive manner in discussion with religious leaders to involve the patients and their relatives into treatment strategies. Lukoff (1985) suggests that good pre-psychotic functioning; acute onset of symptom stressful precipitants and positive exploratory attitudes towards the experience will enable the clinician to differentiate behaviour psychotic episodes and religious experiences.

### Conclusions

There is no doubt that some patients will follow their religious and spiritual beliefs and it is essential that the psychiatrists are honest and open about discussing these with their patients to determine the extent of their beliefs and differentiate these from psychopathology. In addition, the patients must be given every opportunity to explain their beliefs without being blamed or stigmatised. The role of religion in individual's cultural identity must be taken into account when ascertaining mental state and formulating management strategies. To this end getting away from simplistic mind-body dualism will allow the clinicians to plan and offer a holistic management, which may be more acceptable to their patients and carers.

### REFERENCES

Bhugra D (2002): Self concept: Attraction of extreme beliefs and new religious movements. Mental Health Religion & Culture._5, 239-252.

Feibelman J (1963): Men of God and Science. Journal of the Mississippi State Medical Association 15, 29-39.

Greenberg D & Witzum E. (1991): Problems in the treatment of religious patients. American Journal of Psychotherapy 45, 554.

Lukoff D., Lu FG & Turner R (1995): Cultural considerations in the assessment and treatment of religious and spiritual problems. Psychiatric Clinics of North America 18, 467-486

Peterson E & Nelson K (1987): How to meet your clients Spiritual needs? Journal of Psychosocial Nursing_25, 34

